# Supplementation strategies affect the feed intake and performance of grazing replacement heifers

**DOI:** 10.1371/journal.pone.0221651

**Published:** 2019-09-16

**Authors:** Wagner S. Machado, Virginia L. N. Brandao, Valber C. L. Morais, Edenio Detmann, Polyana P. Rotta, Marcos I. Marcondes

**Affiliations:** 1 Animal Science Department, Viçosa Federal University, Av P.H.Rolfs, sn, Dep Zootecnia, Viçosa, Brazil; 2 Department of Animal Sciences, University of Florida, Gainesville, United States of America; Universidade Federal de Mato Grosso do Sul, BRAZIL

## Abstract

The literature lacks studies investigating the performance of supplemented replacement heifers grazing on intensively managed warm-season pasture. Our objective was to evaluate the effects of supplement composition (energetic or protein) on the performance, muscle development, thermogenisis, nutrient intake, and digestibility of replacement Holstein heifers grazing Mombaça grass. Eighteen Holstein heifers with an average age and initial body weight (BW) of 12.57 ± 2.54 mo and 218.76 ±47.6 kg, respectively, were submitted to a randomized block design, with six replicates on a rotational grazing system of *Panicum maximum* cv. Mombaça pasture. Treatments were: control (CON; mineral salt *ad libitum*); energy supplement (ENE; corn meal as supplement, 8% CP and 3.78 Mcal/kg DE); and protein supplement (PRO; corn and soybean meal, 25% CP and 3.66 Mcal/kg DE). Supplements were individually fed at 0.5% BW. The experiment lasted 120 days, subdivided into three periods. Titanium dioxide and indigestible neutral detergent fiber (iNDF) were used to estimate the intakes and digestibility of the nutrients. BW, wither height, thoracic circumference, body length, and ultrasound of ribeye fat thickness measurements were taken once per period. Body condition score (BCS) was assessed twice during the experiment. The MIXED procedure of SAS, including period as a repeated measure, was used and significance was declared at *P* ≤ 0.05. Dry matter intake (DMI), CP intake (CPI) and DE intake were greater in heifers fed PRO compared to CON and ENE. Heifers supplemented with ENE had the lowest DMI. Treatment affected pasture intake/BW; it was similar between PRO and CON heifers, and lower for the ENE treatment. A treatment × period interaction was observed for NDF intake (%BW), in which heifers fed PRO and CON had the greatest NDF intake and ENE had the lowest. The digestibility of DM was the greatest in PRO-supplemented heifers and the lowest in CON heifers. Heifers fed ENE had decreased CP digestibility compared to PRO and CON heifers. Average daily gain (ADG) and thoracic circumference gain were greatest in the PRO treatment. BCS was greater in PRO compared to CON and ENE heifers. Supplementing Holstein heifers at 0.5% BW using PRO supplementation resulted in better animal performance, primarily greater ADG, than feeding ENE or not supplementing (CON). In conclusion, our results indicate that dairy heifers should be fed a protein supplement when grazing intensively managed Mombaça grass pasture.

## Introduction

Raising dairy heifers represents one of the largest annual production costs of a dairy farm operation [1], with feed cost representing up to 60% of the total cost [2]. Therefore, the use of a grazing system is an alternative that can reduce production costs. Modern grazing systems typically use rotational grazing as a way to optimize forage production and animal performance; [3] reported that 85% of the pasture-based dairy farms (in non-organic systems) across the states of New York, Wisconsin and Oregon use a rotational grazing method.

Typically, warm-season grasses are characterized by rapid growth and maturation, which is often associated with lower DM digestibility and animal performance [4], whereas cool season grasses have a lower growth rate and greater quality. When grazing cool season forages, the ADG of dairy heifers can range from 0.84 to 1.18 kg/d without concentrate supplementation [5–7]. On the other hand, when warm-season grasses are managed intensively, NDF can be as low as 58% (%DM), and lignin as low as 2.63% (%DM; [8]). Additionally, the CP content is increased under this condition. For example, [8] reported a value of 18% CP for elephant grass.

Under this management type, animal performance can be limited by the relatively high concentration of protein trapped in the plant cell walls of tropical grasses [9], which necessitates concentrate supplementation to achieve high animal performance. In a study using replacement dairy heifers grazing on warm-season grass (Bermuda grass) under different grazing methods, [10] reported 0.5 kg of ADG regardless of the grazing method; in this study, animals were supplemented with 1.5 kg (approximately 0.6% BW) of concentrate containing corn and soybean meal. In a study using Rhodes grass, [11] reported an ADG of 0.2 kg/d in Friesian and Ayrshire dairy heifers when not supplemented and 0.55 kg/d when supplemented. Nonetheless, the knowledge obtained from studies using cool season grasses does not necessarily directly apply to tropical conditions.

In addition, intake can be physically limited [12] by the relatively high pasture NDF (primarily indigestible NDF) in grazing systems, which is associated with the physical bulk of the forages [13]. In order to meet nutrient requirements, adequate supplementation is crucial to ensure optimal performance and to achieve the target age at first parity [14,15]. The literature lacks studies with replacement heifers grazing on intensively managed warm-season forages such as *Panicum maximum*, and studies that determine supplementation strategies to improve performance under such conditions are warranted.

Therefore, due to the relatively high CP content found in intensively managed warm-season grass, we hypothesized that energy limits animal performance, with ADG being greater in heifers fed an energetic concentrate rather than a protein supplement or no supplementation. Our objective was to evaluate the effects of supplement composition (energetic or protein) on the performance, muscle development, thermogenisis, nutrient intake, and digestibility of replacement Holstein heifers grazing Mombaça grass (*Panicum maximum* cv. Mombaça).

## Material and methods

### Treatments and measurements

This study was conducted in the Dairy Cattle Teaching, Research and Extension unit of the Federal University of Viçosa, Viçosa-MG, Brazil. This study was approved by the ethics committee for animal use at Federal University of Viçosa (Viçosa, MG, Brazil).

Eighteen animals were divided in two blocks and submitted to a completely randomized block design, with a repeated measures scheme. Heifers were considered the experimental unit; therefore, there were six replications per treatment. Heifers were blocked by initial BW, in which the average weight of one group was 188.46 kg (± 56.51 kg) and the other was 239.59 kg (± 27.30 kg), and each nine-animal block grazed a separate set of 15 paddocks. Therefore, blocks were a combination of animals’ weight and set of paddocks and treatments were randomized within each block.

The treatments were: control (CON; no supplementation); energetic supplement (ENE: animals supplemented with corn meal), and protein supplement (PRO: animals fed a mixture containing 49.48% corn and 50.52% soybean meal, DM basis). The chemical composition and characteristics of the pasture and supplements are presented in Tables 1 and 2. Heifers were supplemented at 0.5% BW, and they had *ad libitum* access to mineral mix and water. Individual heifers were fed the same supplement (treatment) during the entire experimental period. Every day at 1200 h, heifers were taken out of the pasture, placed in individual pens and individually fed concentrate. Concentrates were weighted individually for each heifer, according to their adjusted weight, and no orts were allowed. Therefore, amount of supplement was corrected throughout the experiment according to their current BW. Control animals (non-supplemented) were also placed in individual pens, but without supplement.

**Table 1:** Pasture (*Panicum maximum*, cv Mombaça) characteristics, pre and post-grazing sward height of 15 days (average days of cycle) of grazing activities.

Item	Period		
	1^1^	2^2^	3^3^
Accumulated herbage (kg DM/ha/cycle)	1727.34	1485.42	840.12
Accumulated herbage (kg DM/paddock/cycle)	144.41	110.92	82.84
Herbage DM allowance (kg DM/animal/day)	8.02	6.16	5.37
Forage allowance (kg DM of pasture/kg BW)^4^	1.03	0.75	0.35
Grazing efficiency (%)^5^	68.81	87.95	104.25
PreGH^6^ (cm)	75.34	81.51	54.51
PostGH^7^ (cm)	51.96	46.56	33.45

^1^January 14, 2016 to February 22, 2016 (represents the rainy season).

^2^February 23, 2016 to April 2, 2016 (represents the transition between rainy to dray season).

^3^April 3, 2016 to May 12, 2016 (represents the beginning of dry season).

^4^Calculate according to [46]

^5^Calculate as: total DMI (sum of all animals)/accumulated herbage (kg DM/paddock/cycle) × 100

^6^Pre-grazing sward height.

^7^Post-grazing stubble height.

**Table 2:** Pasture and experimental protein (PRO) and energetic (ENE) supplements chemical composition (DM basis).

Item, %DM otherwise stated^1^	Pasture—Period			Supplements	
	1^2^	2^3^	3^4^	PRO	ENE
DM (%)	25.29	28.24	23.65	87.94	89.08
NDF^5^	55.04	59.02	58.84	19.86	12.13
iNDF^6^	8.68	9.90	8.92	0.92	0.95
CP^7^	16.70	15.52	18.11	25.17	8.19
DE (Mcal/kg)^8^	3.08	2.99	3.10	3.66	3.78
Ash	11.18	10.65	10.53	4.24	1.47

^1^Heifers had ad libitum access to mineral. Composition: NaCl = 49.66 g/kg; dicalcium phosphate = 47 g/kg; limestone = 1.71 g/kg; zinc sulphate = 7.25 mg/kg; ferrous sulphate = 4.05 mg/kg; copper sulphate = 2.39 mg/kg; manganese sulphate = 2.15 mg/kg; cobalt sulphate = 0.2 mg/kg; sodium sulphate = 0.16 mg/kg; potassium iodate 0.08 mg/kg.

^2^January 14, 2016 to February 22, 2016 (represents the rainy season).

^3^February 23, 2016 to April 2, 2016 (represents the transition between rainy to dray season).

^4^April 3, 2016 to May 12, 2016 (represents the beginning of dry season).

^5^Neutral detergent fiber.

^6^Indigestable neutral detergent fiber.

^7^Crude protein.

^8^Data estimated based on control animals

On the first day of each experimental period, animals were weighed after 12 h of fasting, and measurements of withers height, thoracic circumference, and body length were taken. To estimate fecal excretion, 10 g/animal/d of titanium dioxide was dosed orally for 8 d starting on d 32, 72, and 112 in the first, second, and third periods, respectively. Three feces samples were collected: at 0600 h on d 37, 1200 h on d 38 and 1800 h on d 39 of each period, therefore feces were collected 3 times per period.

Pasture, supplement and feces samples were analyzed as described below. Samples were oven-dried (55°C) for 72 h and ground to 2 and 1 mm using a mill (Willey, model TE-680, TECNAL, Brazil; [16]) Supplement and feces samples were proportionally mixed during the sampling days and pooled by period and by animal. The 1 mm samples were analyzed for DM ([17]; method 934.01), NDF ([16], INCT-CA method F-002/1), ash ([17]; method 942.05), and CP ([16]; method 990.13). The 2 mm samples were analyzed for indigestible NDF (iNDF). They were also incubated into the rumen of a ruminally-fistulated cow for a period of 288 h, using non-woven textile bags (100 g/m^2^), and NDF was determined from the post-incubation material [18]. For this analysis, a ruminally-fistulated dry cow was used, and fed 80% forage (pasture) and 20% concentrate made of corn and soybean meal, and *ad libitum* mineral mix. Feces were analyzed for titanium dioxide content, according to [16], using the INCT-CA M-007/1 method.

On d 1, 33, 73, and 113, an ultrasound device was used to measure the *gluteus medius* and the *biceps femoris* muscle intercessions, located between the ischial and the ileal tuberosities, by scanning between the 12th and 13th ribs and the rump in the P8 region. We used an 18-cm linear array ultrasound instrument (Aloka SSD-500V, Aloka Co., Ltd., Tokyo, Japan) operated at a frequency of 3.5 MHz. A standoff (Aloka long standoff guide-beef, Aloka Co., Ltd. Tokyo, Japan) and vegetable oil were used for adequate acoustic contact between the transducer, the standoff and the animals’ skin. Ultrasound images were recorded and later analyzed for back fat thickness (BFT) and loin depth (LD) using the BioSoft Toolbox® II for 200 Beef (Biotronics Inc., Ames, Iowa, USA) software. BFT was presented in millimeters, while LD was presented as centimeters squared.

Infrared thermogenic photographs of each animal’s eyes were taken on d 60 and 100 at 1500 h to evaluate the effects of supplementation on heat production [19]. Body condition score (BCS) measurements were taken on the same d as the infrared thermogenic photographs by three trained individuals. BCS was measured on a five-point scale [20], and the average of the three individuals’ measurements was used as the body condition score.

### Grazing management and pasture measurements

The pasture was established two years before the starting date of this experiment. From the establishment of the pasture to the beginning of the experiment, pastures were grazed by dairy heifers using rotational grazing, irrigated and fertilized with 200 kg of N/ha/year and 150 kg of K_2_O/ha/year. The same fertilization procedure was done during the experimental periods, which was manually distributing 20 kg of N/ha and 15 kg of K_2_O/ha in the paddock after each grazing cycle. Aiming to adapt the animals to the experimental procedures and management system, a 45-d adaptation period was used prior to the beginning of the experimental period. The same grazing method, and pre- and post-grazing sward height used during the experimental period was used during these 45 d. During this adaptation period, all heifers were fed the same concentrate containing 18% CP at 0.5% BW (on a DM basis).

The experimental period was from January 14 to May 12 (2016). The experiment was divided into three periods of 40 d each; therefore, the experiment lasted for 120 d. The first period was from January 14 to February 22, which represents rainy period in tropical areas. The second period was from February 23 to April 2, which is considered the transition period between the rainy and dry periods in tropical areas. The third period was from April 3 to May 12, which represents the begging dry period in tropical areas (S1 Fig).

The total experimental area consisted of 30 paddocks of *Panicum maximum* cv. Mombaça, with 816 m^2^ of pasture area and 60 m^2^ of shade in each paddock. Each block containing nine heifers (three from each treatment) had daily access to one fresh paddock and grazed on one group of 15 paddocks, while the second block containing the same number of animals had daily access to a different set of 15 paddocks (S2 Fig). Therefore, each block of heifers grazed one paddock per d.

Paddocks were sized based on the weight of the heaviest block (block 2); therefore, in the case of pasture surplus, put and take animals were used. Based on the previous pasture yield of the same area, we expected a production of approximately 73 kg DM/ha/d during the experimental period. Therefore, for a total of 165 d (45 d for adaptation and 120 d for the experimental period), using 9 heifers/paddock/d, 15 paddocks of 816 m^2^ per block were necessary (considering a grazing efficiency of approximately 65%). The herbage allowance was calculated according to [21], and the target was on average 0.44 kg DM of pasture/kg BW. The actual herbage allowance is presented in Table 1.

**Fig 1:**
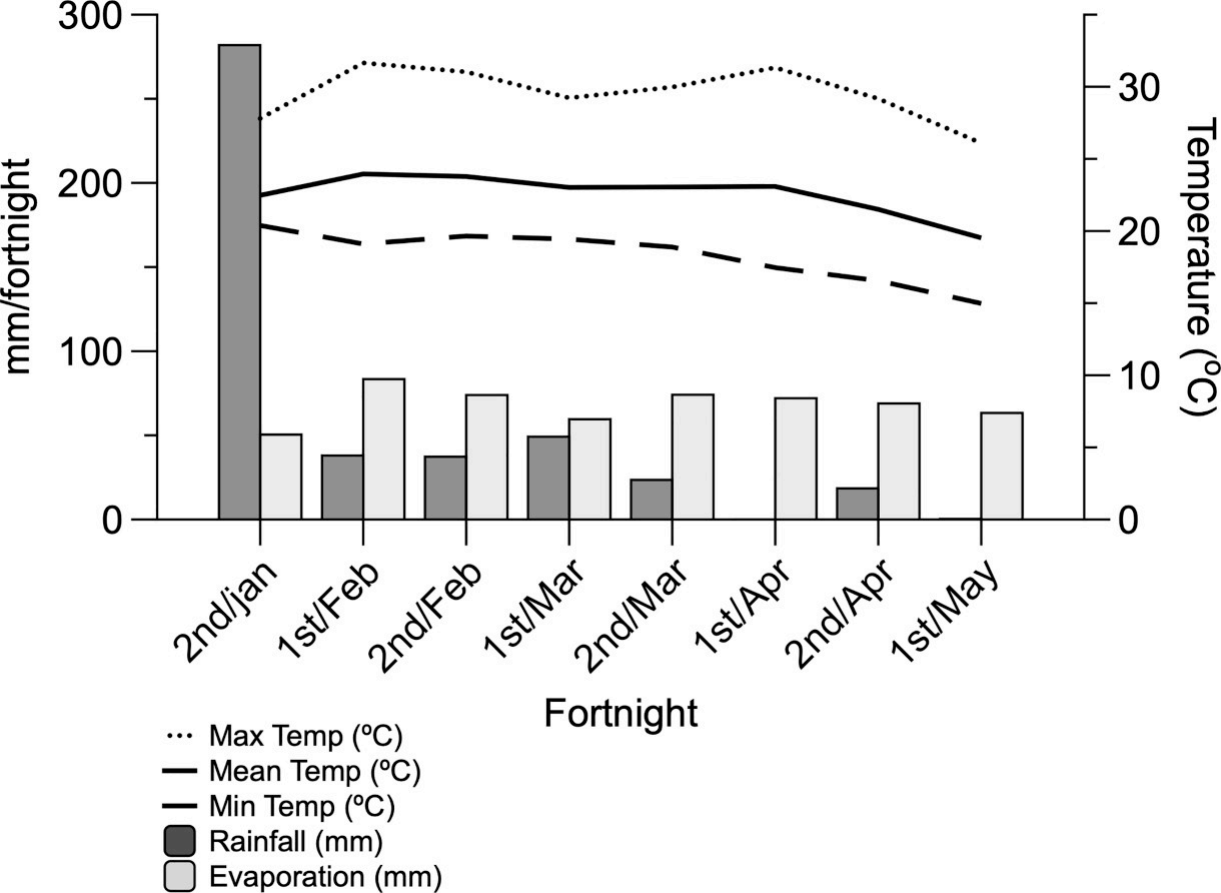
Accumulate fortnightly rainfall and evaporation (mm); minimum, average and maximum (°C) temperatures during the experiment.

The pre-grazing sward height (PreGH) target was 70 cm, and the post-grazing height (PostGH) was 35 cm. When pastures did not reach the target PostGH, put and take heifers with similar BW as the experimental animals were used to reach the target PostGH after the experimental animals were removed from the paddock. An unexpected decrease in rainfall during the third period resulted in decreased herbage accumulation, (Table 1; Fig 1) and put and take heifers were only used during the first and second periods, corresponding to the periods of pasture surplus.

Pre-grazing herbage accumulation was determined in each paddock on the last 7 d of each experimental period. Pasture samples were taken by clippings using a 1.0 x 1.5 m (width x length) exclusion cage. Samples were collected at the actual post-grazing stubble height; therefore, when the target post-grazing height of 35 cm was not achieved, the pasture was sampled at the actual post-grazing height. Furthermore, two exclusion cages were placed in representative areas (based on height and morphological structure) immediately before the beginning of a new grazing cycle in each paddock.

### Statistical analysis

All variables were analyzed using the MIXED procedure of SAS (Statistical Analysis System version 9.4). All data were analyzed as a completely randomized block design, and period was included as a repeated measure in the model:

$$Y_{ijke}=\mu +T_{i}+\delta_{ij}+\beta_{k}+P_{e}+{\left(T\;x\;P\right)}_{ie}+\varepsilon_{ijke}$$

Where μ = general mean; T_i_ = fixed effect of the treatment i; δ_ij_ = random error with a mean of 0 and variance of σ^2^, the variance among animals within treatment, equal to the covariance among repeated measures within animals; β_k_ = random effect of the block j; P_e_ = fixed effect of period; (T x P)_ie_ = fixed effect of the interaction between treatment i and period e; and ε_ijke_ = random error with a mean of 0 and variance of σ^2^, the variance among measures between animals.

The interaction between treatment and block was tested and it was not significant; therefore, it was removed from the model. Seven covariance structures (AR1, CS, UN, TOEP, VC, ARH1, TOEPH) were tested, and CS provided the best fit based on the Akaike information criterion. Means were compared by the least squares method and differences were considered significant when P ≤ 0.05 according to a Student’s t test.

## Results

**Fig 2:**
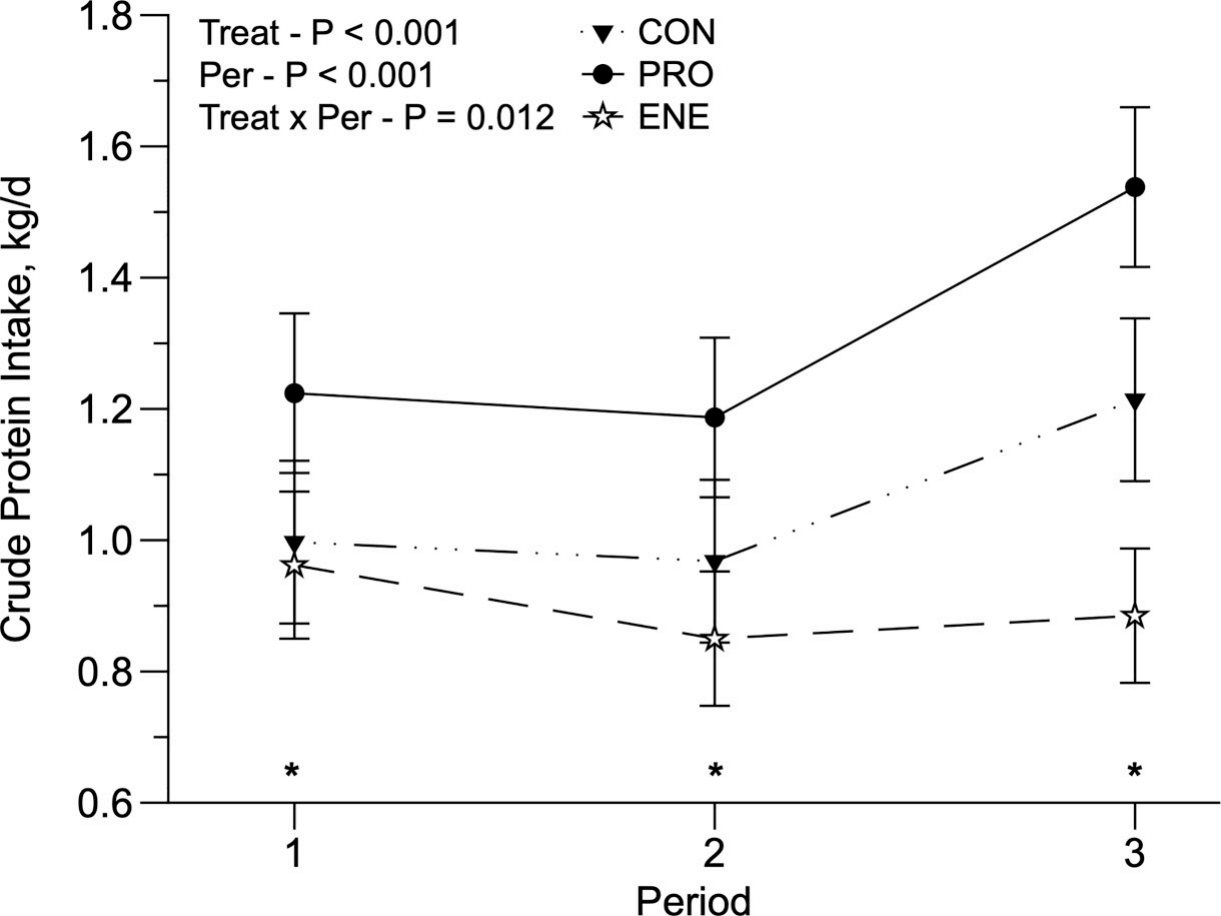
**Crude protein intake of replacement Holstein heifers fed no supplement (CON) or fed protein (PRO) or energy (ENE) supplement on a rotational grazing system of Panicum maximum cv. Mombaça pasture.** *Indicative of significance in the period (P < 0.05).

**Fig 3:**
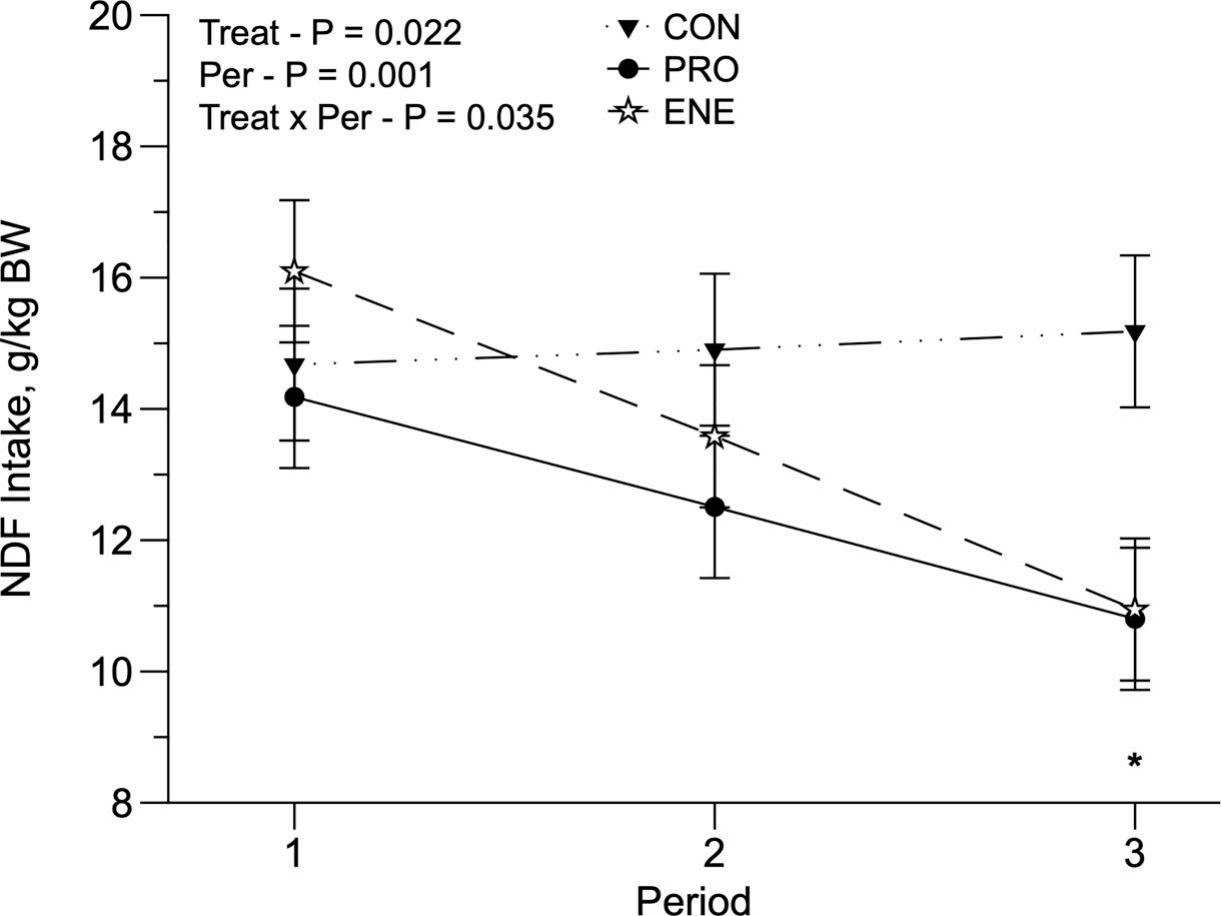
Neutral detergent fiber intake (g/kg BW) of replacement Holstein heifers fed no supplement (CON) or fed protein (PRO) or energy (ENE) supplement on a rotational grazing system of Panicum maximum cv. Mombaça pasture. *Indicative of significance in the period (P < 0.05).

We observed a drastic decrease in rainfall in January; however, the average temperature remained relatively constant throughout the entire experimental period (Fig 1). The accumulated herbage (kg DM/ha/cycle), herbage available per paddock (kg DM/paddock/cycle), herbage allowance (kg DM/animal/day), forage allowance (kg DM of pasture/kg BW), PreGH and PostGH decreased, and grazing efficiency (%) increased throughout the experimental periods (Table 1). Pasture NDF and iNDF were lowest during the first period, and pasture CP was the greatest in the third period (Table 2). We only observed significant interactions (treatment × period) for CP intake (CPI; *P* = 0.012; Fig 2) and NDF intake (NDFI; *P* = 0.035; Fig 3) when expressed as g/kg BW (NDFI/BW). Animals fed PRO had greater CPI in all periods (*P* = 0.012), and in the third period, animals fed ENE had the lowest CPI, compared with CON and PRO animals (Fig 2). Furthermore, CON animals had the greatest NDFI/BW during the third period (Fig 3).

Supplementation strategies affected all intake variables. Animals fed PRO had a greater DMI (*P* = 0.045), pasture intake (*P* = 0.006), NDFI (*P* = 0.012) than ENE (Table 3), and similar to CON. Heifers fed PRO had the greatest DEI. As a consequence, supplement intake was, on average, 1.425 kg/d and 1.081 kg/d for PRO and ENE heifers, respectively. The supplementation strategies also affected pasture intake/BW (*P* = 0.049), in which CON animals had a greater pasture intake/BW, compared to PRO and ENE heifers. A period effect was observed for DMI/BW, pasture intake/BW, CPI/BW, and DEI/BW (*P* < 0.05): they were greater in the first period compared to the second and third periods (Table 3).

**Table 3:** Intake and diet digestibility of replacement Holstein heifers fed no supplement (CON) or fed protein (PRO) or energy (ENE) supplement on a rotational grazing system of *Panicum maximum* cv. Mombaça pasture.

Item	Treatments			Period			SEM	*P*-value		
	CON	PRO	ENE	P1^14^	P2^15^	P3^16^		Treat	Per	Int
DMI^1^ (kg/day)	6.32^ab^	7.17^a^	5.91^b^	6.29	6.41	6.68	0,671	0.045	0.340	0.094
PI^2^ (kg/day)	6.28^a^	5.75^a^	4.83^b^	5.57	5.49	5.79	0.529	0.006	0.528	0.074
NDFI^3^ (kg/day)	3.64^a^	3.57^a^	2.89^b^	3.18	3.38	3.54	0.247	0.012	0.088	0.054
CPI^4^ (kg/day)	1.06	1.32	0.90	1.06	1.01	1.22	0.144	<0.001	<0.001	0.012
DEI^5^ (Mcal/day)	17.8^b^	21.3^a^	17.4^b^	18.6	18.1	19.8	2.235	0.045	0.089	0.300
DMI/BW^6^ (g/kg of BW)	26.1	26.8	28.1	29.0^A^	25.8^B^	26.2^B^	1.413	0.653	0.016	0.168
PI/BW^7^ (g/kg of BW)	26.1^a^	22.0^b^	23.0^b^	25.0^A^	22.0^B^	23.0^B^	0.002	0.049	0.031	0.088
NDFI/BW^8^ (g/kg of BW)	14.9	12.5	13.5	14.9	13.7	12.3	1.123	0.220	0.001	0.035
CPI/BW^9^ (g/kg of BW)	4.20	4.63	4.07	4.88^A^	3.95^B^	4.07^B^	0.203	0.221	<0.001	0.211
DEI/BW^10^ (kcal/kg of BW)	79.2	84.4	79.5	93.1^A^	77.9^B^	72.5^B^	0.030	0.578	0.001	0.070
DMD^11^ (g/kg of BW)	689^b^	723^a^	702^ab^	714^A^	685^B^	714^A^	0.008	0.040	<0.001	0.293
NDFD^12^ (g/kg of BW)	691	696	682	680^B^	670^B^	718^A^	0.007	0.529	<0.001	0.439
CPD^13^ (g/kg of BW)	801^a^	817^a^	761^b^	787^B^	775^B^	816^A^	0.013	<0.001	<0.001	0.407

^1^Total dry matter intake.

^2^Pasture intake.

^3^Neutral detergent fiber intake.

^4^Crude protein intake.

^5^Digestible energy intake (Mcal/day).

^6^Dry matter intake per body weight.

^7^Pasture intake per body weight.

^8^Neutral detergent fiber intake per body weight.

^9^Crude protein intake per body weight.

^10^Digestible energy intake per body weight.

^11^Dry matter digestibility.

^12^Neutral detergent fiber digestibility.

^13^Crude protein digestibility.

^14^January 14, 2016 to February 22, 2016 (represents the rainy season).

^15^February 23, 2016 to April 2, 2016 (represents the transition between rainy to dray season).

^16^April 3, 2016 to May 12, 2016 (represents the beginning of dry season).

^a, b, c^ Values within a row with different superscripts differ significantly among treatments at P<0.05.

^A, B^ Values within a row with different superscripts differ significantly among periods at P<0.05.

Treatments did affect the digestibility of DM (DMD) and CP (CPD; *P* < 0.05; Table 3). Animals fed PRO had greater DMD (*P* = 0.04) compared to CON animals. The ENE supplementation negatively affected CPD, compared to PRO and CON (P = 0.01). Nevertheless, there were period effects for all digestibility variables. The DMD decreased in the second period compared to in the first and third periods. The NDF digestibility (NDFD) and CPD were greater in the third period compared to in the first and second periods. Average daily gain (ADG) and thoracic circumference gain (TCG) were greater for animals fed PRO (*P* < 0.05) compared to CON and ENE (Table 4). Additionally, there was a period effect (*P* < 0.05) on ADG, TCG, and WHG, and a greater performance was observed during the first and second periods compared to the third period (Table 4).

**Table 4:** Performance of replacement Holstein heifers fed no supplement (CON) or fed protein (PRO) or energy (ENE) supplement on a rotational grazing system of *Panicum maximum* cv. Mombaça pasture.

Item	Treatments			Periods			*P*-value		
	CON	PRO	ENE	P1^8^	P2^9^	P3^10^	SEM	Treat	Per	Int
ADG^1^ (kg/d)	0.308^b^	0.570^a^	0.346^b^	0.544^A^	0.512^A^	0.169^B^	0.065	0.007	0.001	0.083
TCG^2^ (cm/d)	0.061^b^	0.110^a^	0.052^b^	0.112^A^	0.087^A^	0.024^B^	0.021	0.030	<0.001	0.253
WHG^3^ (cm/d)	0.056	0.058	0.055	0.079^A^	0.059^A^	0.031^B^	0.008	0.956	0.015	0.080
BLG^4^ (cm/d)	0.096	0.098	0.090	0.138	0.064	0.083	0.025	0.972	0.093	0.290
RFT^5^ (mm)	0.913	0.997	1.028	0.907	1.039	0.991	0.079	0.665	0.254	0.349
BFT^6^ (mm)	1.126	1.368	1.319	1.162	1.309	1.342	0.090	0.163	0.122	0.030
LD^7^ (cm)	48.708	56.676	50.988	47.427	49.690	59.255	5.575	0.140	<0.001	0.027

^1^Average daily gain.

^2^Thoracic circumference gain.

^3^Withers height gain.

^4^Body length gain.

^5^Ribeye fat thickness.

^6^Back fat thickness.

^7^Loin depth.

^8^January 14, 2016 to February 22, 2016 (represents the rainy season).

^9^February 23, 2016 to April 2, 2016 (represents the transition between rainy to dray season).

^10^April 3, 2016 to May 12, 2016 (represents the beginning of dry season).

^a, b, c^ Values within a row with different superscripts differ significantly between supplement strategy at P<0.05.

^A, B^ Values within a row with different superscripts differ significantly among periods at P<0.05.

**Fig 4:**
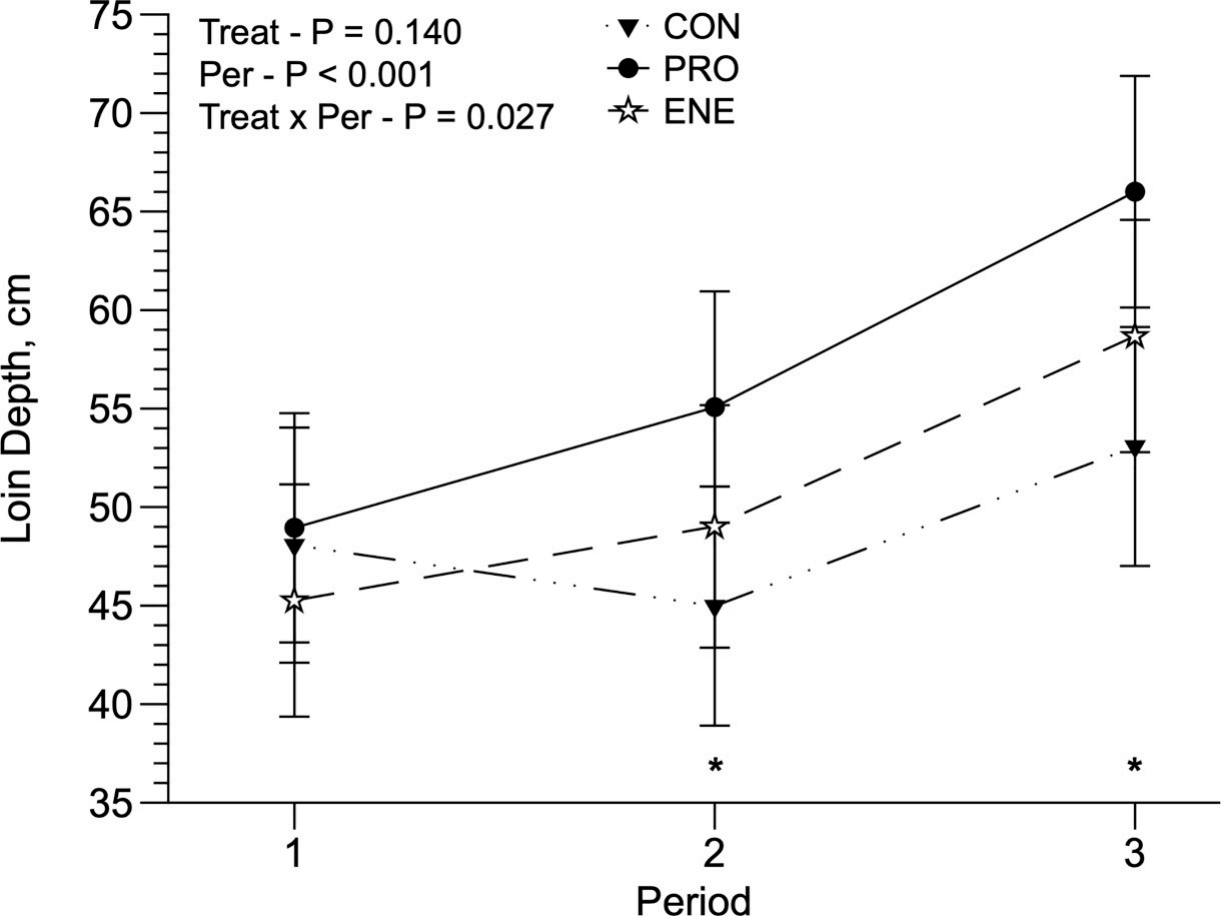
Loin depth of replacement Holstein heifers grazing Panicum maximum cv. Mombaça without supplementation (CON) or fed protein (PRO) or energy (ENE) supplement. *Indicative of significance in the period (P < 0.05).

**Fig 5:**
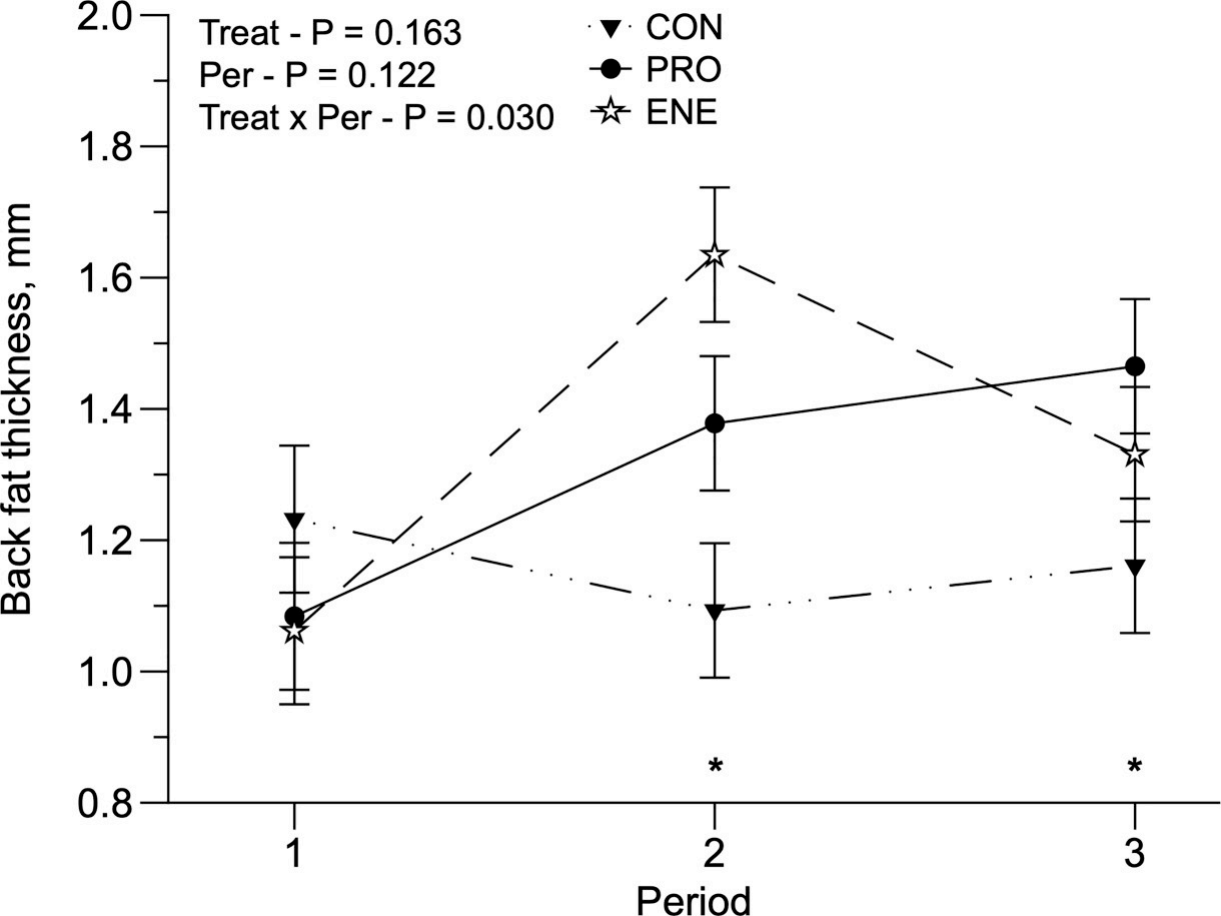
Back fat thickness of replacement Holstein heifers grazing Panicum maximum cv. Mombaça without supplementation (CON) or fed protein (PRO) or energy (ENE) supplement. *Indicative of significance in the period (P < 0.05).

There was a treatment × period interaction for LD and BFT. The LD was greater (*P* = 0.027) in PRO-fed animals, but only during the second and third periods, compared to CON animals (Fig 4). BFT was greater (*P* = 0.03) in ENE compared to CON animals in the second period, and PRO animals had greater BFT than CON animals in the third period (Fig 5). Treatments did not influence ribeye fat thickness (RFT). Treatments did not affect the animals’ eye temperature, although in the third period all temperature measurements were lower (P < 0.01; Table 5). BCS was greater in PRO animals (3.429 ± 0.113; *P* = 0.02) compared to CON and ENE animals (3.238 and 3.037 ± 0.113, respectively).

**Table 5:** Eye temperature and body score condition (BSC) of replacement Holstein heifers fed no supplement (CON) or fed protein (PRO) or energy (ENE) supplement on a rotational grazing system of *Panicum maximum* cv. Mombaça pasture.

Item	Treatments			Day			*P*-value		
	CON	PRO	ENE	d 60^5^	d 100^6^	SEM	Treat	Day	Int
ETmax^1^ (°C)	37.043	37.325	37.550	38.682^A^	35.930^B^	0.486	0.276	<0.001	0.692
ETmin^2^ (°C)	34.786	35.416	35.241	36.680^A^	33.600^B^	0.371	0.483	<0.001	0.887
ETavg^3^ (°C)	36.139	36.416	36.483	37.709^A^	34.983^B^	0.423	0.533	<0.001	0.723
BCS^4^	3.238^b^	3.429^a^	3.037^c^	3.212^B^	3.250^A^	0.113	0.026	0.531	0.766

^1^Maximum eye temperatures

^2^Minimum eye temperatures

^3^Average eye Temperatures

^4^Body condition score, five-point scale

^5^Medial day between 1st and 2nd period

^6^Medial day between 2nd and 3rd period.

^a, b, c^ Values within a row with different superscripts differ significantly between supplement strategy at P<0.05

^A, B^ Values within a row with different superscripts differ significantly between period at P<0.05.

Substitution rates are presented in Table 6. Overall, during periods one and two the substitution rate was 2.2 folds greater on ENE fed animals, comparing with PRO animals. While during the third period the substitution was 11 folds greater on ENE fed animals, comparing with PRO animals.

**Table 6:** Substitution rates of replacement of Holstein heifers fed protein (PRO) or energy (ENE) supplement on a rotational grazing system of *Panicum maximum* cv. Mombaça pasture.

Period	Substitution rate (%)^1^	
	PRO	ENE
1^2^	0.27	0.60
2^3^	0.53	1.21
3^4^	0.17	1.88

^1^Calculated as follows: Substitution rate (kg/kg) = (pasture intake in control animals–pasture intake in supplemented animals)/supplement intake (Bargo et al., 2003). SR > 1 indicates that total DMI was lower in supplemented animals comparing to control animals.

^2^January 14, 2016 to February 22, 2016 (represents the rainy season).

^3^February 23, 2016 to April 2, 2016 (represents the transition between rainy to dray season).

^4^April 3, 2016 to May 12, 2016 (represents the beginning of dry season).

## Discussion

As rainfall decreased across the experimental periods (Fig 1), herbage accumulation also decreased (Table 1). Decreasing precipitation and temperature results in reduced water availability in the soil, decreasing pasture growth [22]. This condition affects herbage total accumulation, and the number of tillers and new leaves [22]. As a consequence of weather conditions, sward height during the third period was below the target pre-grazing height and heifers grazed on pasture greater in NDFD, resulting in a grazing efficiency that was close to 100% (Table 1). Grazing efficiency represents the proportion of pasture consumed by all animals compared with the total herbage accumulation. Therefore, a grazing efficiency above 100% indicates that the animals consumed pasture below the target stubble height, which was 35 cm in the present study. During the third period when post-grazing height was 33.45 cm (Table 1), the grazing efficiency was greater than 104%.

The target pre-grazing height was determined based on studies using 95% light interception by the canopy as the ideal physiological state to start a grazing event [23–24]. This stage is correlated to sward height, and below the target height, there is a greater accumulation of leaves and better pasture quality; however, animal performance is limited by low pasture herbage accumulation [25]. This was observed during the third period of the present study, in which the pasture had greater DM, NDF, and CP digestibility (Table 3). Even though the forage nutritive value was improved during the third period, which could result in greater ADG, the reduced forage quantity was a factor that limited the animal response. Forage nutritive value explains most of the variation in ADG only when forage quantity is not limiting [26].

Protein supplementation resulted in lower DMI of ENE supplemented animals. Heifers fed PRO had increased DEI, which was a reflection of greater CPI and DMD (Table 3), ultimately increasing ADG (Table 4). Similar results were reported by [27] when supplementing Holstein x Zebu steers grazing *Brachiaria decumbens* with increasing levels of protein supplementation. Heifers fed the PRO treatment had greater CPI in all three periods compared to the CON and ENE treatment (Fig 2). This result, associated with greater DMI, DEI, and NDFI observed in heifers fed PRO compared to ENE and CON, strengthens our hypothesis that PRO treatment stimulates pasture intake. Additionally, ENE fed animals had a lower substitution rate when compared to PRO, resulting in lower CPI from pasture intake. Nevertheless, feeding cows high protein diets such as PRO might increase nitrogen excretion [28], and while a greater performance may be achieved, it is at an environmental cost [29].

Pasture substitution by a supplement is usually greater when energetic supplements are supplied [30–32], as was observed in our study (Table 6). The greater substitution rate observed in the animals fed ENE (Table 6) is likely the main reason for the observed effects on pasture intake and NDFI. Pasture intake was greater for animals fed PRO compared to ENE, and they also had greater NDFI. Interestedly, even though there was an increase in pasture CP/BW content during the third period (Table 2), animals fed ENE did not increase their CPI (Fig 2); this is likely a reflection of the greater substitution rate of the ENE-fed animals, during the third period (Table 6). On the other hand, animals fed PRO increased their CPI due to greater protein intake coming from the supplement (25% CP), with no changes to pasture intake. The effect of pasture substitution by concentrate can be minimized by protein supplementation [33–34], an effect that was confirmed in the present study.

As a result of the greater energy density in the ENE treatment, DEI and DMI did not differ between ENE and CON, despite the greater pasture intake of heifers fed the CON treatment. The PRO animals consumed more digestible energy and had greater CPD than the CON animals, probably for the same reason (the nutrient concentration provided by the supplement). The high energy density from corn in the ENE supplement might have resulted in a decrease in pasture intake (greater substitutive effect), which was reflected in similar DEI values between the CON and ENE treatments (Table 3). When the pasture has a sufficient CP, greater animal performance may be limited by the energy density of the diet [35–36] and the supplement can be formulated with low-protein content feedstuffs, such as corn meal. Nevertheless, the interaction between supplement and pasture intake should be carefully monitored to control situations where supplemented animals decrease their DMI even when the grass has a high nutritive value, as observed for the ENE animals in the third period (Table 6).

The CP from the ENE supplement (Table 2) and the observed substitution rates (Table 6) may elucidate the reason for the lack of a difference in DEI between the CON and ENE, as well as explain why CPI was greater for CON than ENE animals. Supplements based on corn meal have a lower protein input coming from the supplement and a different amino acid profile than soybean-based concentrates, which can change the ruminal fermentation end-products. By providing corn meal, there might be a greater proliferation of amylotic bacteria and greater supply of soluble carbohydrates, which can reduce ruminal fiber degradation. On the other hand, by feeding a PRO supplement, it is possible to favor the passage of ruminal fiber [36–38] by increasing the concentration of branched-chain VFA and the synthesis of microbial protein.

Furthermore, supplements typically have greater digestible energy than pasture [37]. Comparing the CON and ENE treatments, CON heifers compensated for the absence of supplementation with greater pasture intake, and ENE heifers had a decreased pasture intake, consequently decreased DEI from pasture. This lower DEI was compensated by the high energy in the supplement, balancing their DEI when compared with CON heifers. Additionally, CPI was greater in PRO-fed animals during all three periods (Fig 2), and no differences between CON and ENE were observed in the first and the second periods; however, the CPI of the ENE-fed animals was less than the CON animals in the third period. The substitution rate of the ENE treatment was greater than 1 during second and third periods (1.21 and 1.88 respectively; Table 6), which is likely a consequence of the decreased herbage allowance during these periods (Table 1).

We hypothesized that ENE supplementation would stimulate pasture digestion, leading to an increase in pasture intake; however, our results do not confirm this hypothesis. When nutrient intake was expressed as a percentage of BW, only pasture intake/BW was affected by the treatments, and it was greater for heifers fed the CON treatment compared to the ENE and PRO treatments. Control animals were not supplemented; therefore, they required a greater pasture intake/BW to meet their daily nutrient requirements. A similar effect was found by [39] in animals fed exclusively hay, who had a greater hay intake (%BW) compared to animals provided energy or protein supplementation. Furthermore, PRO was not different from ENE in terms of pasture intake/BW, probably because of a greater body weight gain (Table 4), which decreased the proportion of pasture intake per kg of BW since substitution rates in PRO were not as intense as in ENE (Table 6). [39] reported greater hay DMI/BW for the control group (as described above); according to their data, the supplemented groups decreased hay DMI/BW by 33% when compared with the control groups. In our study, the decrease in pasture intake (%BW) was 15.32% and 11.53% for the PRO and ENE supplementations, respectively, and PRO was not different from CON (P > 0.05).

There was a treatment x period interaction for NDFI/BW (Fig 3). The main difference occurred in the third period, where the CON had a greater NDFI/BW compared to PRO and ENE supplementation. During the third period, there was a decline in herbage accumulation, which was reflected in a greater NDFD due to the lower PreGH. A possible explanation could be the pasture substitution rate of PRO and ENE, and the fact that non-supplemented heifers (CON) had a greater pasture intake/BW.

Heifers fed PRO had a greater DMD compared to CON heifers. [40] reported supplementation as the main factor associated with increases in DMD. Therefore, because in the present study we did not observe a difference in NDFD between the CON and PRO treatments, supplement intake is likely responsible for the increase in DMD in the PRO compared to the CON animals. On the other hand, the CPD differences in the ENE compared with the PRO and CON treatments might be due to the ruminal fermentation condition that resulted from ENE supplementation. A greater proliferation of non-fiber carbohydrate-fermenting microorganisms may increase the need for nitrogen [38]. When nitrogen availability in the rumen is not in synchrony, it could result in a nitrogen limitation for microbial protein synthesis, which may affect the digestibility of nutrients [35,36,41]. In addition, the literature lacks data for the DMD and CPD of supplemented dairy heifers grazing on intensively managed Mombaça pasture. [39] observed an increase in organic matter digestibility when rumen degraded protein increased from 61.17% to 69.73%. Therefore, the CPD was greater in the PRO supplementation strategy compared with ENE, but PRO did not differ from the CON. It is possible that the greater CPD of the CON compared to ENE is mainly due to changes in the ruminal microbial population in the heifers fed ENE [37,41].

The larger supply of amino acids in the PRO diet, associated with greater energy intake, resulted in a greater ADG and TCG in PRO animals (Table 4). [35] observed a linear increase in ADG in crossbred steers fed up to 24% CP in the supplement. According to [35], animals fed a protein supplement had greater ADG because of the synchronism between ruminal fermentable organic matter and nitrogen utilization by rumen microorganisms for microbial growth, associated with greater amino acid availability for intestine absorption. Our results and those of [35] demonstrate that it is possible to obtain better performance and body development when a protein supplement is fed to heifers grazing on high quality tropical pastures, such as Mombaça pastures. In addition, CON and ENE heifers did not reach an ADG of 0.7 to 0.8 kg/d recommended for Holstein heifers, which can compromise mammary gland development and future lactations [28,42,43]. Nevertheless, this satisfactory gain of PRO animals was achieved only in Periods 1 and 2, which indicates that during the dry season (Period 3) achieving adequate rates of gain might require greater levels of supplementation for grazing heifers.

Due to lower pasture growth in the third period (Table 1), ADG, TCG, and WHD also decreased (Table 4), which likely limited animal performance. There was a treatment x period interaction for BFT, which occurred mainly in the second and third periods (Fig 5). During the second period, BFT differences were observed between CON and ENE animals, and was greater in ENE animals. During the third period, heifers fed PRO had greater BFT than CON heifers. It is possible that, in the first two periods, animals fed ENE had more fat deposition than protein, which may be a result of the lack of nitrogen in the rumen, associated with a greater energy supply. In the third period, the decline in pasture availability may have led to the use of this deposited fat to meet requirements. On the other hand, PRO and CON animals had an almost constant deposition of BFT, but it was greater for PRO animals, for the reasons discussed above. Another possible explanation for the BFT results of animals fed PRO is the additional protein supply associated with greater DMD during the third period, which may have resulted in greater ruminal organic acid production, including precursors for fat synthesis [44,45]. There was a treatment x period interaction for LD, which happened in the second and third periods (Fig 4). The LD differences only occurred between PRO and CON, and it was greater in PRO during both periods (P < 0.05). These differences may be due to the PRO-fed animals having a greater CPI in all periods. Protein supplementation may support greater protein synthesis in animal muscles [46], which was reflected in a greater LD in PRO animals.

[20] demonstrated that ocular temperature may be related to an animal’s heat production. We hypothesized that PRO animals, due to greater protein deposition, would present greater heat production; however, treatments did not affect body temperature, and only a period effect was observed. The lower body temperature on d 100 was related to the lower environmental temperature (Fig 1). Maximum and minimum day temperatures were 31 and 28.4°C, and 17.5 and 13.5°C on d 60 and 100, respectively. The air temperature at the moment of measurement was 30°C and 27.6°C on d 60 and 100, respectively. Although the environmental temperature helped to explain the lower ocular temperature on d 100, additional studies are warranted to evaluate the interaction between ocular temperature, as measured by a thermal infrared camera, and grazing animals’ heat production.

In summary, heifers fed the PRO supplement had better body development, with greater intake, compared to ENE heifers, and greater CPI, DEI, ADG and TCG than CON and ENE heifers. When comparing the PRO supplement with the CON and ENE supplements, a better performance was noted in the heifers fed the PRO supplement. PRO supplementation resulted in a better association between forage and supplement intake. Therefore, our results indicate that dairy heifers should be fed a protein supplement when grazing intensively managed Mombaça grass pasture. Nonetheless, future studies should focus on the best protein level and amino acid profile in the supplement provided for Holstein heifers grazing highly intensive warm-season pasture.

## Supporting information

S1 Fig Lineout of the experiment’s days and samplings. (DOCX)

S2 Fig Experiment map. Animals were blocked by weight, so block 1 grazed the green area and block 2 grazed the light blue area. Animals colored in black represent CON treatment, animals colored in red represent PRO treatment, and animals colored in blue represent ENE treatment. Every day all animals were placed in the yellow area for individual supplement feeding.
(DOCX)

S1 Data (XLSX)
