# Supplementary figures and images for: Supplementation strategies affect the feed intake and performance of grazing replacement heifers
Source: PLoS One. 2019 Sep 16;14(9):e0221651. doi: 10.1371/journal.pone.0221651 (PMC6746373; doi:10.1371/journal.pone.0221651)

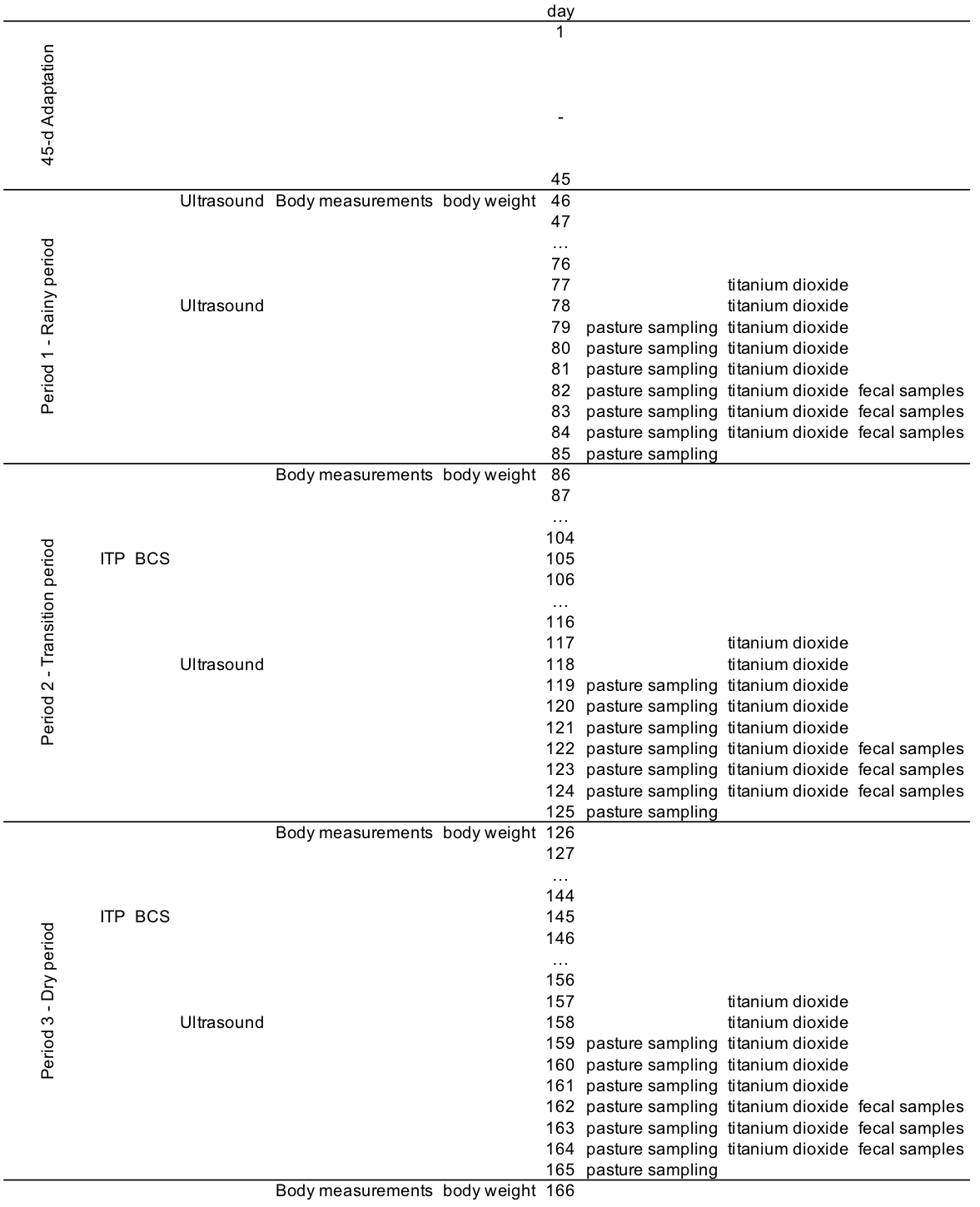


**S1 Fig. Lineout of the experiment’s days and samplings**

Supplement: S1 Fig — (DOCX) [file pone.0221651.s001.docx]
