# Supplementary material for: Supplementation strategies affect the feed intake and performance of grazing replacement heifers
Source: PLoS One. 2019 Sep 16;14(9):e0221651. doi: 10.1371/journal.pone.0221651 (PMC6746373; doi:10.1371/journal.pone.0221651)
Supplement: S2 Fig — Animals were blocked by weight, so block 1 grazed the green area and block 2 grazed the light blue area. Animals colored in black represent CON treatment, animals colored in red represent PRO treatment, and animals colored in blue represent ENE treatment. Every day all animals were placed in the yellow area for individual supplement feeding. (DOCX) [file pone.0221651.s002.docx]

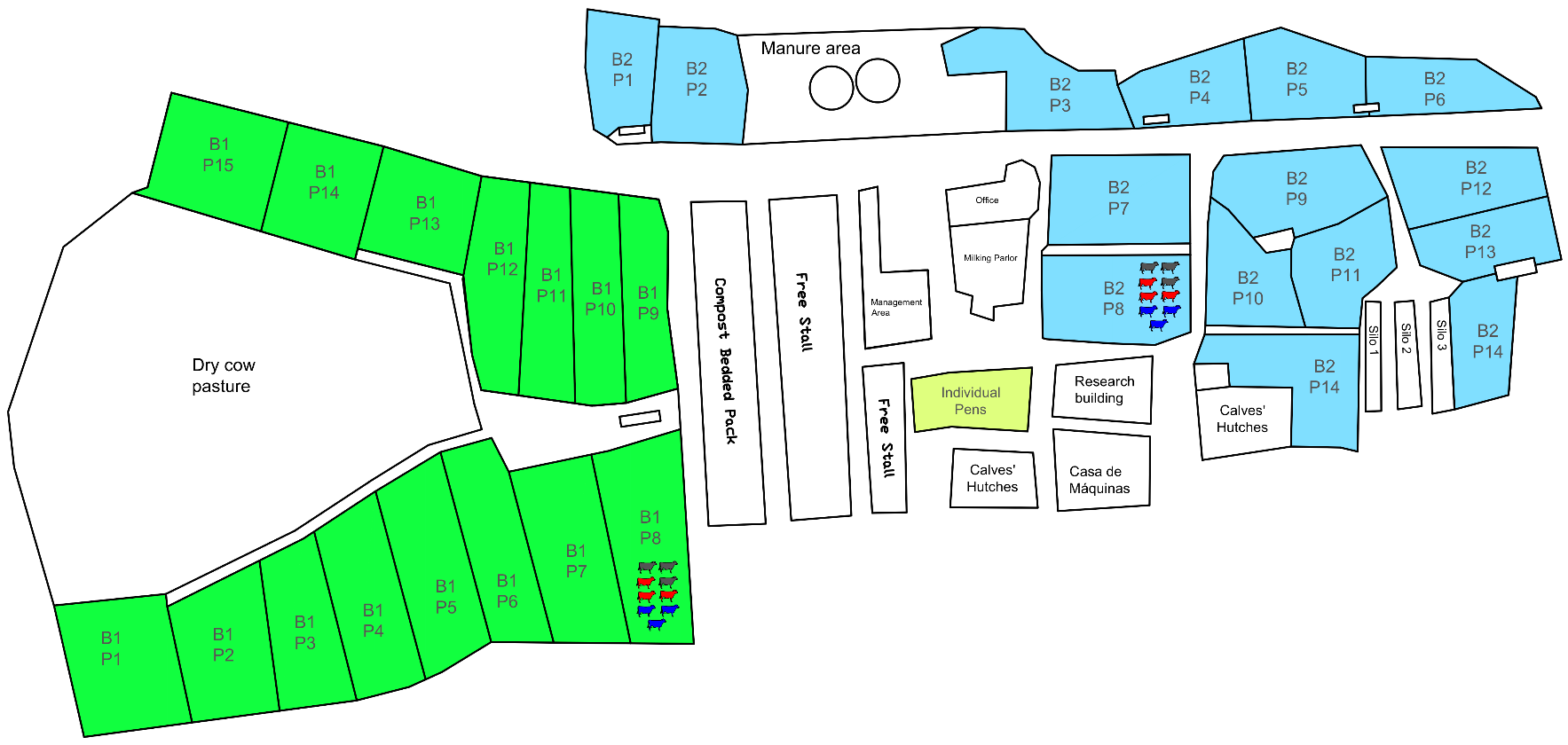


**S2 Fig. Experiment map. Animals were blocked by weight, so block 1 grazed the green area and block 2 grazed the light blue area. Animals colored in black represent CON treatment, animals colored in red represent PRO treatment, and animals colored in blue represent ENE treatment. Every day all animals were placed in the yellow area for individual supplement feeding.**
